# A Delay Learning Algorithm Based on Spike Train Kernels for Spiking Neurons

**DOI:** 10.3389/fnins.2019.00252

**Published:** 2019-03-27

**Authors:** Xiangwen Wang, Xianghong Lin, Xiaochao Dang

**Affiliations:** College of Computer Science and Engineering, Northwest Normal University, Lanzhou, China

**Keywords:** spiking neural networks, supervised learning, spike train kernels, delay learning, synaptic delays

## Abstract

Neuroscience research confirms that the synaptic delays are not constant, but can be modulated. This paper proposes a supervised delay learning algorithm for spiking neurons with temporal encoding, in which both the weight and delay of a synaptic connection can be adjusted to enhance the learning performance. The proposed algorithm firstly defines spike train kernels to transform discrete spike trains during the learning phase into continuous analog signals so that common mathematical operations can be performed on them, and then deduces the supervised learning rules of synaptic weights and delays by gradient descent method. The proposed algorithm is successfully applied to various spike train learning tasks, and the effects of parameters of synaptic delays are analyzed in detail. Experimental results show that the network with dynamic delays achieves higher learning accuracy and less learning epochs than the network with static delays. The delay learning algorithm is further validated on a practical example of an image classification problem. The results again show that it can achieve a good classification performance with a proper receptive field. Therefore, the synaptic delay learning is significant for practical applications and theoretical researches of spiking neural networks.

## 1. Introduction

Spiking neural networks (SNNs) that composed of biologically plausible spiking neurons are usually known as the third generation of artificial neural networks (ANNs) (Maass, 1997). The spike trains are used to represent and process the neural information in spiking neurons, which can integrate many aspects of neural information, such as time, space, frequency, and phase, etc. (Whalley, 2013; Walter et al., 2016). As a new brain-inspired computational model of the neural network, SNN has more powerful computing power compared with a traditional neural network model (Maass, 1996). SNNs can simulate all kinds of neural signals and arbitrary continuous functions, which are very suitable for processing the brain neural signals (Ghosh-Dastidar and Adeli, 2009; Beyeler et al., 2013; Gütig, 2014).

Supervised learning for SNNs refers to that for multiple given input spike trains and desired output spike trains, finding an appropriate synaptic weight matrix of the SNNs in order to assimilate the actual output spike trains of output neurons to the corresponding desired output spike trains, that is, the value of the error evaluation function between them is the smallest. Researchers have proposed many supervised multi-spike learning algorithms for spiking neurons in recent years (Lin et al., 2015b). The basic ideas of these algorithms mainly include gradient descent, synaptic plasticity, and spike train convolution.

Supervised learning algorithms based on gradient descent use gradient computation and error back-propagation for adjusting the synaptic weights, and ultimately minimize the error function that indicates the deviation between the actual and desired output spike trains. Xu et al. (2017) proposed a supervised learning algorithm for spiking neurons based on gradient descent, in which an online adjustment mechanism is used. The basic idea of supervised learning algorithms based on synaptic plasticity is using the mechanism of synaptic plasticity caused by the timing correlation of spike trains of presynaptic and postsynaptic neurons to design the supervised learning rules. Representative algorithms are the remote supervised method (ReSuMe) (Ponulak and Kasiński, 2010) and its extensions (Lin et al., 2016, 2018). Supervised learning algorithms based on spike train convolution are constructed by the inner products of spike trains (Paiva et al., 2009; Park et al., 2013). Discrete spike trains are firstly converted to continuous functions through the convolution calculation of the specific kernel function, and then constructing the supervised learning algorithm for SNNs. The adjustment of synaptic weights depends on the convolved continuous functions corresponding to spike trains, which can realize the learning of the spatio-temporal pattern of the spike trains. Representative algorithms are spike pattern association neuron (SPAN) (Mohemmed et al., 2012), precise-spike-driven (PSD) (Yu et al., 2013), and the work of Lin et al. (Lin et al., 2015a; Wang et al., 2016; Lin and Shi, 2018).

Experimental research (Minneci et al., 2012) proves that synaptic delays widely exist in biological neural networks. The time delay has an effect on the processing ability of the nervous system (Xu et al., 2013). At present, in most supervised learning algorithms for SNNs, only the connection strength, namely the synaptic weight between pre- and post-synapse, is adjusted. Neuroscientific studies have shown that the synaptic delays in the biological nervous system are not always invariant, but can be modulated (Lin and Faber, 2002; Boudkkazi et al., 2011). However, efficient synaptic delay learning algorithms are few. In recent years, researchers have introduced the delay learning to ReSuMe learning rule (Ponulak and Kasiński, 2010) and proposed some ReSuMe-based delay learning algorithms (Taherkhani et al., 2015a,b, 2018; Guo et al., 2017). Simulation results show that the delay versions of ReSuMe achieve learning accuracy and learning speed improvements compared with the original ReSuMe. Shrestha et al. (Shrestha and Song, 2016) formulated an adaptive learning rate scheme for delay adaptation in the SpikeProp algorithm (Bohte et al., 2002) based on delay convergence analysis. Simulation results of spike train learning show that the extended algorithm improves learning performance of the basic SpikeProp algorithm. There are also some other delay learning algorithms (Napp-Zinn et al., 1996; Wang et al., 2012; Hussain et al., 2014) have been proposed, and further implemented by hardware.

In this paper, we propose a new supervised delay learning algorithm based on spike train kernels for spiking neurons, in which both the synaptic weights and the synaptic delays can be adjusted. The rest of this paper is organized as follows. In section 2, we first introduce the spiking neuron model and the kernel representation of the spike train used in this paper and then derive the supervised learning rules of both synaptic weights and synaptic delays using gradient descent method. A series of spike train learning tasks and an image classification task are performed to test and verify the learning performance of our proposed learning algorithm in section 3. The discussion of our proposed algorithm is presented in section 4. Finally, we conclude this paper in section 5.

## 2. Materials and Methods

### 2.1. Spiking Neuron and Spike Train Representation

#### 2.1.1. Spike Response Model

The short-term memory spike response model (SRM) (Gerstner and Kistler, 2002) is employed in delay learning. It expresses the membrane potential *u* at time *t* as an integral over the past, including a model of refractoriness. In the short-term memory SRM, only the last fired spike $t_{o}^{l}$ contributes to the refractoriness. Assuming that a neuron has *N*_*I*_ input synapses, the *i*th synapse transmits a total of *N*_*i*_ spikes and the *f*th spike (*f* ∈ [1, *N*_*i*_]) is fired at time $t_{i}^{f}$. The internal state *u*(*t*) of the neuron at time *t* is given by:

(1) $$\begin{array}{l} u\left(t\right)=\overset{N_{I}}{\underset{i=1}{\sum }}\overset{N_{i}}{\underset{f=1}{\sum }}w_{i}\varepsilon \left(t-t_{i}^{f}-d_{i}\right)+\eta \left(t-t_{o}^{l}\right) \end{array}$$

where *w*_*i*_ and *d*_*i*_ are the synaptic weight and the synaptic delay for the *i*th synapse, respectively. When the internal state variable *u*(*t*) crosses the firing threshold θ, the neuron fires a spike.

**Figure 1:**
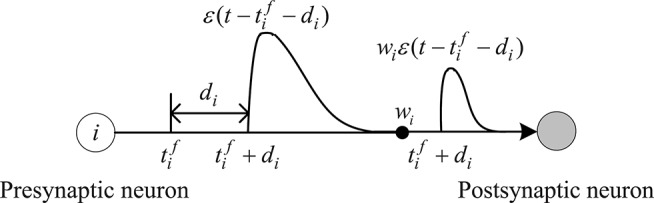
Spike response function.

The spike response function $\varepsilon \left(t-t_{i}^{f}-d_{i}\right)$ describes the effect of the presynaptic spike on the internal state of the postsynaptic neuron, as shown in Figure 1. It is expressed as:

(2) $$\begin{array}{l} \varepsilon (t-t_{i}^{f}-d_{i})=\{\begin{array}{lll} \frac{t-t_{i}^{f}-d_{i}}{\tau }\exp(1-\frac{t-t_{i}^{f}-d_{i}}{\tau }) & , & t-t_{i}^{f}-d_{i}>0\\ 0 & , & t-t_{i}^{f}-d_{i}\leq 0 \end{array} \end{array}$$

where τ indicates the time decay constant of postsynaptic potentials, which determines the shape of the spike response function.

In addition, $\eta \left(t-t_{o}^{l}\right)$ is the refractoriness function, which is mainly reflected in the effect that only the last output spike $t_{o}^{l}$ contributes to the refractoriness:

(3) $$\begin{array}{l} \eta (t-t_{o}^{l})=\{\begin{array}{lll} -\theta \exp(-\frac{t-t_{o}^{l}}{\tau_{R}}) & , & t-t_{o}^{l}>0\\ 0 & , & t-t_{o}^{l}\leq 0 \end{array} \end{array}$$

where θ is the neuron threshold. τ_*R*_ is the time constant, which determines the shape of refractoriness function. When $t-t_{o}^{l}\in \left(0,\infty \right)$, the refractoriness function $\eta \left(t-t_{o}^{l}\right)$ is negative. When $t-t_{o}^{l}\to 0$, the minimum value of $\eta \left(t-t_{o}^{l}\right)$ is −θ. When $t-t_{o}^{l}\to \infty$, the value of $\eta \left(t-t_{o}^{l}\right)$ is gradually increased to 0.

#### 2.1.2. Spike Train and Its Kernel Representation

The spike train *s* = {*t*^*f*^ ∈ Γ:*f* = 1, ⋯ , *N*} represents the ordered sequence of spike times fired by the spiking neuron in the time interval Γ = [0, *T*], and can be expressed formally as:

(4) $$\begin{array}{l} s\left(t\right)=\overset{N}{\underset{f=1}{\sum }}\delta \left(t-t^{f}\right) \end{array}$$

where *t*^*f*^ is the *f*th spike time in *s*(*t*), *N* is the number of spikes in *s*(*t*), and δ(·) represents the Dirac delta function, δ(*x*) = 1 if *x* = 0 and δ(*x*) = 0 otherwise. Considering the synaptic delay in the input spike train, the input spike train *s*_*i*_(*t* − *d*_*i*_) with synaptic delay is defined as:

(5) $$\begin{array}{l} s_{i}\left(t-d_{i}\right)=\overset{N_{i}}{\underset{f=1}{\sum }}\delta \left(t-t_{i}^{f}-d_{i}\right) \end{array}$$

where $t_{i}^{f}$ is the *f*th spike in the input spike train *s*_*i*_(*t* − *d*_*i*_), *d*_*i*_ is the synaptic delay between presynaptic neuron *i* and postsynaptic neuron, and *N*_*i*_ is the number of spikes in *s*_*i*_(*t* − *d*_*i*_).

In order to facilitate the analysis and calculation, we can choose a specific kernel function κ(·), using the convolution to convert the discrete spike train to a continuous function:

(6) $$\begin{array}{l} f_{s}\left(t\right)=s\left(t\right)*\kappa \left(t\right)=\overset{N}{\underset{f=1}{\sum }}\kappa \left(t-t^{f}\right) \end{array}$$

Therefore, the convolved continuous functions corresponding to the input spike train *s*_*i*_(*t* − *d*_*i*_), actual output spike train *s*_*o*_(*t*), and desired output spike train *s*_*d*_(*t*) can be expressed as follows according to Equation (6):

(7) $$\begin{array}{l} f_{s_{i}}\left(t-d_{i}\right)=s_{i}\left(t-d_{i}\right)*\kappa \left(t\right)=\sum_{f=1}^{N_{i}}\kappa \left(t-t_{i}^{f}-d_{i}\right) \end{array}$$

(8) $$\begin{array}{l} f_{s_{o}}\left(t\right)=s_{o}\left(t\right)*\kappa \left(t\right)=\sum_{h=1}^{N_{o}}\kappa \left(t-t_{o}^{h}\right) \end{array}$$

(9) $$\begin{array}{l} f_{s_{d}}\left(t\right)=s_{d}\left(t\right)*\kappa \left(t\right)=\sum_{g=1}^{N_{d}}\kappa \left(t-t_{d}^{g}\right) \end{array}$$

where $t_{i}^{f}$, $t_{o}^{h}$, and $t_{d}^{g}$ are spikes in *s*_*i*_(*t* − *d*_*i*_), *s*_*o*_(*t*), and *s*_*d*_(*t*), respectively. *N*_*i*_, *N*_*o*_, and *N*_*d*_ are numbers of spikes in *s*_*i*_(*t* − *d*_*i*_), *s*_*o*_(*t*), and *s*_*d*_(*t*), respectively.

In SNNs, neural information or external stimuli is encoded into spike trains. The computation performed by a single spiking neuron can be defined as a mapping from the presynaptic spike trains to the appropriate postsynaptic spike train. In order to analyze the relationship between the presynaptic and postsynaptic spike trains, we use linear-nonlinear Poisson (LNP) model (Schwartz et al., 2006), in which the spiking activity of the postsynaptic neuron is defined by the estimated intensity functions of the presynaptic neurons. Some researches show that the relationship between the postsynaptic spike train *s*_*o*_(*t*) and the contributions of all presynaptic spike trains *s*_*i*_(*t* − *d*_*i*_) can be expressed as a linear relationship for excitatory synapse through the convolved continuous functions (Cash and Yuste, 1999; Carnell and Richardson, 2005):

(10) $$\begin{array}{l} f_{s_{o}}\left(t\right)=\overset{N_{I}}{\underset{i=1}{\sum }}w_{i}f_{s_{i}}\left(t-d_{i}\right) \end{array}$$

where *w*_*i*_ represents the synaptic weight between the presynaptic neuron *i* and the postsynaptic neuron, and *N*_*I*_ is the number of presynaptic neurons.

### 2.2. Learning Rules Based on Spike Train Kernels

**Figure 2:**
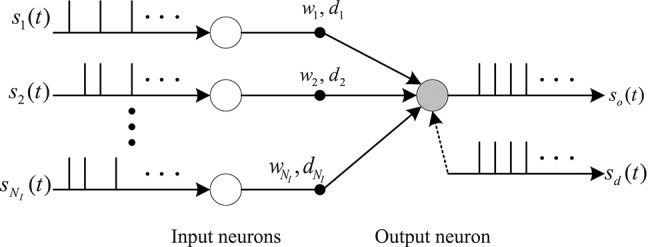
Network structure of neurons with synaptic delays.

In this section, we use the gradient descent method to deduce the learning rule of synaptic weights and delays. We consider a fully connected feed-forward network structure of spiking neurons as shown in Figure 2. There are *N*_*I*_ input neurons and one output neuron in this model. There is only one synaptic connection between an input neuron and an output neuron. Each synapse has a connection weight *w*_*i*_ and a time delay *d*_*i*_. The aim of the delay learning method is to train the neuron to produce a desired output spike train *s*_*d*_(*t*) in response to multiple spatio-temporal input spike patterns *s*_*i*_(*t* − *d*_*i*_). In the synaptic delay learning model, both the synaptic weight *w*_*i*_ and the synaptic delay *d*_*i*_ are adjusted to train the output neuron to fire the actual output spike train *s*_*o*_(*t*) toward the desired output spike train *s*_*d*_(*t*).

Defining the error function of the network is an important prerequisite for supervised learning of spiking neurons. The instantaneous error for the network can be formally defined in terms of the square difference between the convolved continuous functions *f*_*s*_*o*__(*t*) and *f*_*s*_*d*__(*t*) corresponding to the actual output spike train *s*_*o*_(*t*) and desired output spike train *s*_*d*_(*t*) at time *t*. It can be represented as:

(11) $$\begin{array}{l} E\left(t\right)=\frac{1}{2}{\left[f_{s_{o}}\left(t\right)-f_{s_{d}}\left(t\right)\right]}^{2} \end{array}$$

So, the total error of the network in the time interval Γ is $E=\int_{\Gamma }E\left(t\right)dt$.

#### 2.2.1. Learning Rule of Synaptic Weights

According to the gradient descent rule, the change of synaptic weight Δ*w*_*i*_ from the presynaptic neuron *i* to the postsynaptic neuron is computed as follows:

(12) $$\begin{array}{l} \Delta w_{i}=-\eta \nabla E_{w} \end{array}$$

where η is the learning rate of synaptic weights and ∇*E*_*w*_ is the gradient of the spike train error function *E* for the synaptic weight *w*_*i*_. The gradient can be expressed as the integration of the derivative of the instantaneous error *E*(*t*) with respect to synaptic weight *w*_*i*_ in the time interval Γ:

(13) $$\begin{array}{l} \nabla E_{w}=\int_{\Gamma }\frac{\partial E\left(t\right)}{\partial w_{i}}dt \end{array}$$

Using the chain rule, the derivative of the error function *E*(*t*) at time *t* to synaptic weight *w*_*i*_ can be represented as the product of two partial derivative terms:

(14) $$\begin{array}{l} \frac{\partial E\left(t\right)}{\partial w_{i}}=\frac{\partial E\left(t\right)}{\partial f_{s_{o}}\left(t\right)}\frac{\partial f_{s_{o}}\left(t\right)}{\partial w_{i}} \end{array}$$

According to Equation (11), the first partial derivative term of the right-hand part of Equation (14) is computed as:

(15) $$\begin{array}{l} \frac{\partial E\left(t\right)}{\partial f_{s_{o}}\left(t\right)}=\frac{\partial \left[\frac{1}{2}{\left[f_{s_{o}}\left(t\right)-f_{s_{d}}\left(t\right)\right]}^{2}\right]}{\partial f_{s_{o}}\left(t\right)}=f_{s_{o}}\left(t\right)-f_{s_{d}}\left(t\right) \end{array}$$

According to Equation (10), the second partial derivative term of the right-hand part of Equation (14) is computed as:

(16) $$\begin{array}{l} \frac{\partial f_{s_{o}}\left(t\right)}{\partial w_{i}}=\frac{\partial \left[\sum_{i=1}^{N_{I}}w_{i}f_{s_{i}}\left(t-d_{i}\right)\right]}{\partial w_{i}}=f_{s_{i}}\left(t-d_{i}\right) \end{array}$$

Therefore, the gradient ∇*E*_*w*_ in Equation (13) can be computed as follows according to Equations (15 and 16):

(17) $$\begin{array}{l} \nabla E_{w}=\int_{\Gamma }\left[f_{s_{o}}\left(t\right)-f_{s_{d}}\left(t\right)\right]f_{s_{i}}\left(t-d_{i}\right)dt \end{array}$$

On the basis of the deduction process discussed above, a supervised learning rule of synaptic weights based on spike train kernels for spiking neurons with synaptic delays is given. The learning rule of the synaptic weights is expressed as follows:

(18) $$\begin{array}{l} \Delta w_{i}=-\eta \nabla E_{w}=\eta \int_{\Gamma }\left[f_{s_{d}}\left(t\right)-f_{s_{o}}\left(t\right)\right]f_{s_{i}}\left(t-d_{i}\right)dt \end{array}$$

According to Equations (7–9), the synaptic weights learning can be further rewritten as:

(19) $$\begin{array}{l} \Delta w_{i}=\eta \left[\overset{N_{d}}{\underset{g=1}{\sum }}\overset{N_{i}}{\underset{f=1}{\sum }}\kappa \left(t_{d}^{g}-t_{i}^{f}-d_{i}\right)-\overset{N_{o}}{\underset{h=1}{\sum }}\overset{N_{i}}{\underset{f=1}{\sum }}\kappa \left(t_{o}^{h}-t_{i}^{f}-d_{i}\right)\right] \end{array}$$

The learning rate η has a great influence on the convergence speed of the learning process, which can directly affect the training time and the training accuracy. Here we define an adaptive adjustment method of learning rate according to the firing rate of actual output spike train of neurons. Firstly, a scaling factor β is defined according to the different firing rates of the spike train. It is assumed that the firing rate of the spike train of neurons is *r*, and the referenced firing rate range is [*r*_*min*_, *r*_*max*_]. When *r* ∈ [*r*_*min*_, *r*_*max*_], the scaling factor is β = 1; otherwise, the expression of β is:

(20) $$\begin{array}{l} \beta =\{\begin{array}{lll} \frac{r_{min}-r}{r_{max}-r_{min}} & , & r<r_{min}\\ \frac{r-r_{max}}{r_{max}-r_{min}} & , & r>r_{max} \end{array} \end{array}$$

The learning rate in the referenced firing rate range is called the referenced learning rate η^*^, and its value is the best learning rate for a given firing rate range. According to the scaling factor β and the referenced learning rate η^*^ in the firing rate range, the adaptive adjustment method of learning rate is:

(21) $$\begin{array}{l} \eta =\{\begin{array}{lll} (1+\beta )\eta^{*} & , & r<r_{min}\\ \eta^{*} & , & r_{min}\leq r\leq r_{max}\\ \eta^{*}/(1+\beta ) & , & r>r_{max} \end{array} \end{array}$$

#### 2.2.2. Learning Rule of Synaptic Delays

Here we derive the learning rule of synaptic delays with the similar derivation of synaptic weights. The synaptic delay change Δ*d*_*i*_ from the presynaptic neuron *i* to the postsynaptic neuron is computed as follow:

(22) $$\begin{array}{l} \Delta d_{i}=-\alpha \nabla E_{d} \end{array}$$

where α is the learning rate of synaptic delays and ∇*E*_*d*_ is the gradient of the spike train error function *E* for the synaptic delay *d*_*i*_. The gradient can be expressed as the integration of the derivative of the instantaneous error *E*(*t*) with respect to synaptic delay *d*_*i*_ in the time interval Γ:

(23) $$\begin{array}{l} \nabla E_{d}=\int_{\Gamma }\frac{\partial E\left(t\right)}{\partial d_{i}}dt \end{array}$$

Using the chain rule, the derivative of the error function *E*(*t*) to synaptic delay *d*_*i*_ at time *t* can be calculated as the product of two partial derivative terms:

(24) $$\begin{array}{l} \frac{\partial E\left(t\right)}{\partial d_{i}}=\frac{\partial E\left(t\right)}{\partial f_{s_{o}}\left(t\right)}\frac{\partial f_{s_{o}}\left(t\right)}{\partial d_{i}} \end{array}$$

According to Equations (7 and 10), the second partial derivative term of the right-hand part of Equation (24) is computed as:

(25) $$\begin{array}{l} \frac{\partial f_{s_{o}}\left(t\right)}{\partial d_{i}}=\frac{\partial \left[\sum_{i=1}^{N_{I}}w_{i}f_{s_{i}}\left(t-d_{i}\right)\right]}{\partial d_{i}}\\ =\frac{\partial \left[\sum_{i=1}^{N_{I}}w_{i}\sum_{f=1}^{N_{i}}\kappa \left(t-t_{i}^{f}-d_{i}\right)\right]}{\partial d_{i}}\\ =w_{i}\frac{\partial \left[\sum_{f=1}^{N_{i}}\kappa \left(t-t_{i}^{f}-d_{i}\right)\right]}{\partial d_{i}} \end{array}$$

For simplicity, here we choose the Laplacian kernel function to convert spike trains. It is defined as:

(26) $$\begin{array}{l} \kappa \left(s\right)=\exp\left(-\frac{\left|s\right|}{\tau }\right) \end{array}$$

where τ is the scale parameter of the Laplacian kernel function. So the partial derivative term of the right-hand part of Equation (25) is computed as:

(27) $$\begin{array}{l} \begin{array}{c} \frac{\partial \left[\sum_{f=1}^{N_{i}}\kappa \left(t-t_{i}^{f}-d_{i}\right)\right]}{\partial d_{i}}=\frac{\partial \left[\sum_{f=1}^{N_{i}}\exp\left(-\frac{\left|t-t_{i}^{f}-d_{i}\right|}{\tau }\right)\right]}{\partial d_{i}}\\ =\frac{1}{\tau }\overset{N_{i}}{\underset{f=1}{\sum }}\exp\left(-\frac{\left|t-t_{i}^{f}-d_{i}\right|}{\tau }\right)\\ =\frac{1}{\tau }f_{s_{i}}\left(t-d_{i}\right) \end{array} \end{array}$$

Therefore, on the basis of Equations (15), (25), and (27), the derivative ∂*E*(*t*)/∂*d*_*i*_ in Equation (23) can be further rewritten as:

(28) $$\begin{array}{l} \frac{\partial E\left(t\right)}{\partial d_{i}}=\frac{1}{\tau }w_{i}\left[f_{s_{o}}\left(t\right)-f_{s_{d}}\left(t\right)\right]f_{s_{i}}\left(t-d_{i}\right) \end{array}$$

According to the deduction process discussed above, a supervised learning rule of synaptic delays based on spike train kernels for spiking neurons with Laplacian kernel is given. The learning rule of the synaptic delays is expressed as follows:

(29) $$\begin{array}{l} \Delta d_{i}=-\alpha \nabla E_{d}=\alpha \frac{1}{\tau }w_{i}\int_{\Gamma }\left[f_{s_{d}}\left(t\right)-f_{s_{o}}\left(t\right)\right]f_{s_{i}}\left(t-d_{i}\right)dt \end{array}$$

According to Equations (7–9), the learning rule of synaptic delays can be further rewritten as:

(30) $$\begin{array}{l} \Delta d_{i}=\alpha \frac{1}{\tau }w_{i}\left[\overset{N_{d}}{\underset{g=1}{\sum }}\overset{N_{i}}{\underset{f=1}{\sum }}\kappa \left(t_{d}^{g}-t_{i}^{f}-d_{i}\right)-\overset{N_{o}}{\underset{h=1}{\sum }}\overset{N_{i}}{\underset{f=1}{\sum }}\kappa \left(t_{o}^{h}-t_{i}^{f}-d_{i}\right)\right] \end{array}$$

### 2.3. Supervised Learning Algorithm for Spiking Neurons

Algorithm 1 represents the training process of spike train learning using our proposed supervised learning rule. In the beginning, we initialize all parameters of SNNs, mainly including the spiking neuron model and its parameters, the input and desired output spike trains, the synaptic weights and delays. Secondly, we calculate the actual output spike train of the output neuron according to the input spike trains and the spiking neuron model and then calculate the spike train error of the output neuron according to the actual and desired output spike train. Finally, we adjust all synaptic weights and delays according to our proposed learning rules of synaptic weights and delays. This process is called a learning epoch. Repeating the training process until the network error *E* = 0 or the upper limit of learning epochs is exceeded, the training process is ended.

**Algorithm 1:**
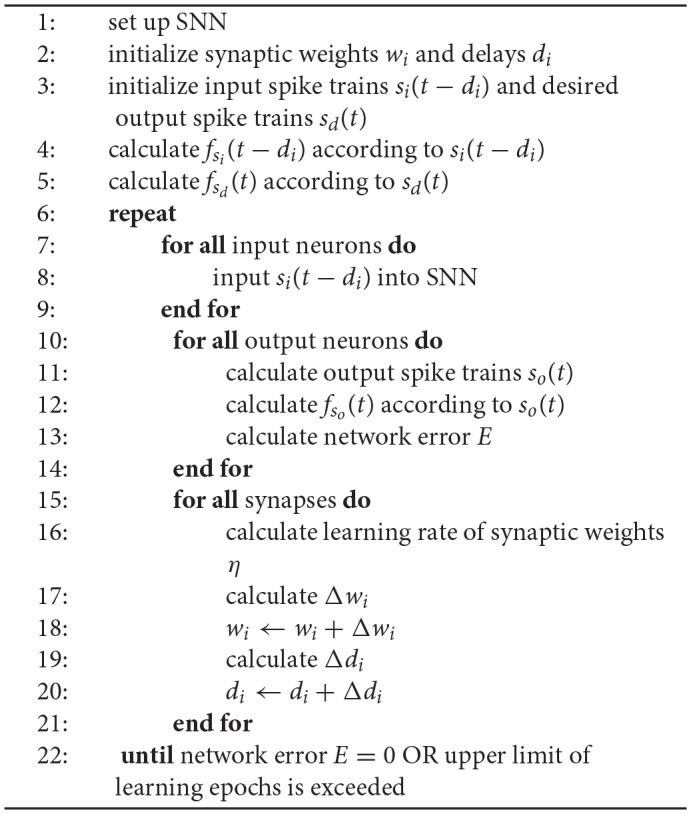
supervised learning algorithm for spiking neurons

## 3. Results

In this section, a series of spike train learning experiments and an image classification task are presented to demonstrate the learning capabilities of our proposed learning algorithm. At first, we analyze the learning process of our proposed algorithm. Then, we analyze the effects of the parameters of synaptic delays on learning performance, such as the learning rate of synaptic delays, the maximum allowed synaptic delays and the upper limit of learning epochs. In addition, we also analyze the effects of the parameters of network simulation on learning performance, such as the number of synaptic inputs, the firing rate of spike trains and the length of spike trains, and compare with the network with static synaptic delays on learning performance. Finally, we use the proposed delay learning algorithm to solve an image classification problem and compare with some other supervised learning algorithms for spiking neurons.

### 3.1. Parameter Settings and Learning Evaluation

Our experiments run on Java 1.7 on a quad-core system with 4-GB RAM in a Windows 10 environment. We use the clock-driven simulation strategy with time-step *dt* = 0.1ms to implement the spike train learning tasks. All reference parameters are shown in Table 1. Initially, the synaptic weights and the synaptic delays are generated as the uniform distribution in the interval [*w*_*min*_, *w*_*max*_] and [*d*_*min*_, *d*_*max*_], respectively. Every input spike train and desired output spike train is generated randomly by a homogeneous Poisson process within the time interval of Γ with firing rate *r*_*in*_ and *r*_*out*_, respectively. Except for the learning process of spike trains demonstrated in section 3.2.1 and the image classification problem presented in section 3.3, the all simulation results are averaged over 100 trials, and on each testing trial, the learning algorithm is applied for a maximum of 500 learning epochs or until the network error *E* = 0. In the training process, the learning rate of synaptic weights is adjusted adaptively. The spiking neurons are described by the short-term memory SRM. The Laplacian kernel function κ(*s*) = exp(−|*s*|/τ) with parameter τ = 10 is used in all simulations.

**Table 1:** Reference parameters in the simulation.

**Parameters**		**Identifiers**	**Value**
	Number of input neurons	*N*_*I*_	500
	Number of output neurons	*N*_*O*_	1
Network simulation	Firing rate of input spike trains	*r*_*in*_	20 Hz
	Firing rate of desired output spike trains	*r*_*out*_	50 Hz
	Length of spike trains	Γ	200 ms
	Time constant of postsynaptic potential	τ	2 ms
SRM neuron model	Time constant of refractory period	τ_*R*_	50 ms
	Spike firing threshold	θ	1
	Length of the absolute refractory period	*t*_*R*_	1 ms
	Minimum synaptic weights	*w*_*min*_	0
Synaptic weights	Maximum synaptic weights	*w*_*max*_	0.5
	Referenced learning rate of synaptic weights	η^*^	0.005
	Minimum synaptic delays	*d*_*min*_	0 ms
Synaptic delays	Maximum synaptic delays	*d*_*max*_	15 ms
	Learning rate of synaptic delays	α	3

To quantitatively evaluate the learning performance, we use the spike train kernels to define a measure *C* to express the distance between the desired output spike train *s*_*d*_(*t*) and the actual output spike train *s*_*o*_(*t*), which is equivalent to the correlation-based metric *C* (Schreiber et al., 2003). The metric is calculated after each learning epoch according to:

(31) $$\begin{array}{l} C=\frac{\langle f_{s_{d}}\left(t\right),f_{s_{o}}\left(t\right)\rangle }{∥f_{s_{d}}\left(t\right)∥∥f_{s_{o}}\left(t\right)∥} \end{array}$$

where 〈*f*_*s*_*d*__(*t*), *f*_*s*_*o*__(*t*)〉 is the inner product of *f*_*s*_*d*__(*t*) and *f*_*s*_*o*__(*t*). $\|f_{s_{d}}(t)\|=\sqrt{\langle f_{s_{d}}(}t),f_{s_{d}}(t)\rangle$ and $\|f_{s_{o}}(t)\|=\sqrt{\langle f_{s_{o}}(}t),f_{s_{o}}(t)\rangle$ are the Euclidean norms of convolved continuous functions corresponding to spike trains *s*_*d*_(*t*) and *s*_*o*_(*t*), respectively. In order to keep in line with the measure described in Schreiber et al. (2003), here we use the Gaussian filter function to convert the spike trains. Measure *C* = 1 for identical spike trains and decreases toward 0 for loosely correlated spike trains.

### 3.2. Learning Sequences of Spikes

#### 3.2.1. Analysis of the Learning Process

**Figure 3:**
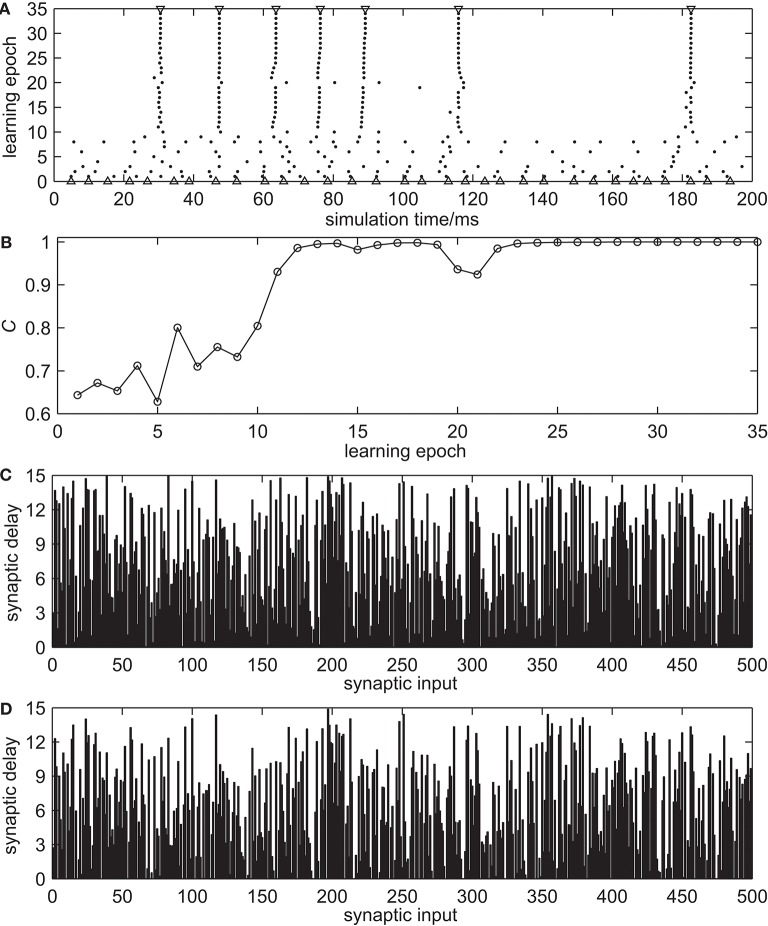
The spike train learning process of proposed synaptic delay learning algorithm. **(A)** The complete learning process. △, the initial actual output spike train before learning; ▽, desired output spike train; •, actual output spike trains during the learning process. **(B)** The evolution of learning accuracy with measure *C*. **(C)** The synaptic delays before learning. **(D)** The synaptic delays after learning.

Figure 3 demonstrates the spike train learning process of one trial using the proposed synaptic delay learning rule to reproduce the desired output spatio-temporal spike pattern. Figure 3A shows the complete learning process in the time interval Γ, which includes the desired output spike train, the initial output spike train before learning and the actual output spike trains during the learning process. It can be seen that the actual output spike trains are closer to the desired output spike train during the learning process. The evolution of learning accuracy with measure *C* during the learning process is presented in Figure 3B. During the learning process, especially in the early stage, dithering occurs easily. However, the learning accuracy *C* increases gradually. After 30 learning epochs, the learning accuracy *C* reached 1.0. The synaptic delays before and after learning are shown in Figures 3C,D, respectively. These learning results show that the spiking neuron can successfully learn the desired output spike train using the proposed synaptic delay learning algorithm.

#### 3.2.2. Parametric Analysis of Synaptic Delays

**Figure 4:**
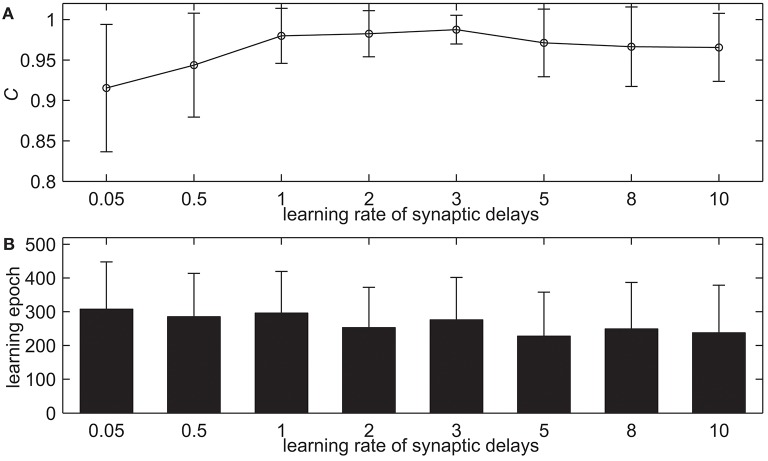
The learning results with the different learning rates of synaptic delays α after 500 learning epochs. **(A)** The learning accuracy *C*. **(B)** The learning epochs when the learning accuracy *C* reaches the maximum value.

Here we test our proposed delay learning algorithm with the different learning rates of synaptic delays α, the maximum allowed synaptic delays *d*_*max*_ and the upper limit of learning epochs. Figure 4 shows the learning results of delay learning algorithm with the different learning rates of synaptic delays α. The α takes 0.05, 0.5, 1.0, 2.0, 3.0, 5.0, 8.0, 10.0 in total of eight values. The learning accuracy with measure *C* after 500 learning epochs is shown in Figure 4A. It can be seen that the measure *C* increases slightly when α increases gradually. When α = 3.0, the learning accuracy is *C* = 0.9874. When α increases further, the measure *C* decreases slightly, in addition, the standard deviation increased. When α = 8.0, the learning accuracy is *C* = 0.9664. Figure 4B shows the learning epochs when the learning accuracy *C* reaches the maximum value. From Figure 4B we can see that when α increases gradually, the learning epochs do not change too much. When α = 3.0, the mean learning epoch is 276.07. When α = 8.0, the mean learning epoch is 249.14. This simulation indicates that the proposed delay learning algorithm can well learn with the different learning rates of synaptic delays in a large range. In the rest of the simulations, the learning rate of synaptic delays is α = 3.0.

**Figure 5:**
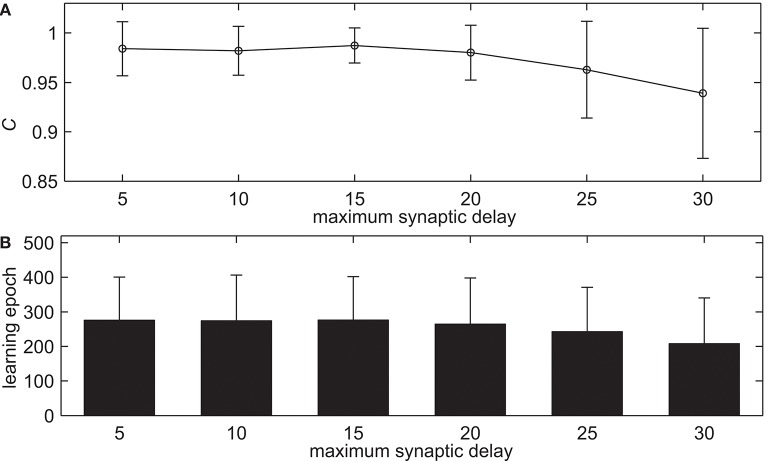
The learning results with the different maximum allowed synaptic delays *d*_*max*_ after 500 learning epochs. **(A)** The learning accuracy *C*. **(B)** The learning epochs when the learning accuracy *C* reaches the maximum value.

Neuroscience experiments give evidence to the variability of synaptic delay values, from 0.1 to 44 ms (Swadlow, 1992; Toyoizumi et al., 2005; Paugam-Moisy et al., 2008). This simulation tests the proposed delay learning algorithm with the different maximum allowed synaptic delays *d*_*max*_, the learning results are shown in Figure 5. *d*_*max*_ increases from 5 to 30 ms with an interval of 5 ms. Figure 5A shows the learning accuracy with measure *C* after 500 learning epochs. From Figure 5A we can see that the delay learning algorithm can learn with high learning accuracy. The learning accuracy *C* basically remains the same when *d*_*max*_ less than 20 ms. When *d*_*max*_ increases further, the learning accuracy decreases, in addition, the standard deviation is increasing. For example, when *d*_*max*_ = 10 ms, the learning accuracy is *C* = 0.9821. When *d*_*max*_ = 25 ms, the learning accuracy is *C* = 0.9629. Figure 5B shows the learning epochs when the learning accuracy *C* reaches the maximum value. It can be seen that the learning epochs do not change too much when *d*_*max*_ increases gradually. For example, when *d*_*max*_ = 10 ms, the mean learning epoch is 274.06. When *d*_*max*_ = 25 ms, the mean learning epoch is 242.68. This simulation indicates that the proposed delay learning algorithm can learn from different maximum synaptic delays *d*_*max*_ in a large range. It is robust for various synaptic delays. In the rest of the simulations, the maximum synaptic delays is *d*_*max*_ = 15 ms.

**Figure 6:**
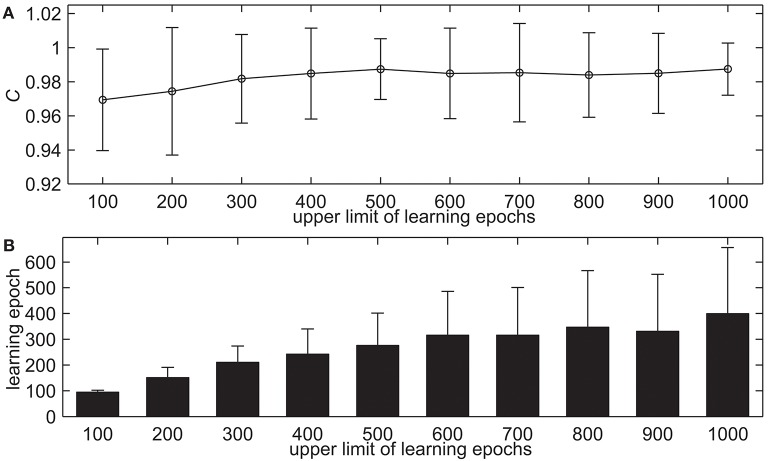
The learning results with the different upper limit of learning epochs. **(A)** The learning accuracy *C*. **(B)** The learning epochs when the learning accuracy *C* reaches the maximum value.

The upper limit of learning epochs is a relatively important evaluation factor for supervised learning. If the upper limit of learning epochs is too small, the network cannot be fully trained, which will lead to the problem that the model cannot solve problems well. Conversely, if the upper limit of learning epochs is too large, it will take too much time to train the network. In this simulation, we test the proposed delay learning algorithm with the different upper limit of learning epochs, the learning results are shown in Figure 6. The upper limit of learning epochs increases from 100 to 1, 000 with an interval of 100, while the other settings remain the same. Figure 6A shows the learning accuracy with measure *C*. It can be seen that in the beginning, the learning accuracy *C* increases when the upper limit of learning epochs increases gradually. When the upper limit of learning epochs increases further, the learning accuracy *C* does not change too much. For example, when the upper limit of learning epochs is 400, the learning accuracy is *C* = 0.9849. When the upper limit of learning epochs is 800, the learning accuracy is *C* = 0.9850. Figure 6B shows the learning epochs when the learning accuracy *C* reaches the maximum value. From Figure 6B we can see that when the upper limit of learning epochs increases gradually, the actual learning epochs increase. When the upper limit of learning epochs is 600, the mean learning epoch is 315.78. When the upper limit of learning epochs increases further, the actual learning epochs do not change too much, but the standard deviation is increasing. When the upper limit of learning epochs is 900, the mean learning epoch is 330.98. This simulation indicates that the proposed delay learning algorithm can learn with high learning accuracy, and increasing the upper limit of learning epochs cannot significantly improve learning accuracy. In the rest of the simulations, the upper limit of learning epochs is 500.

#### 3.2.3. Comparative Analysis With Static Synaptic Delays

**Figure 7:**
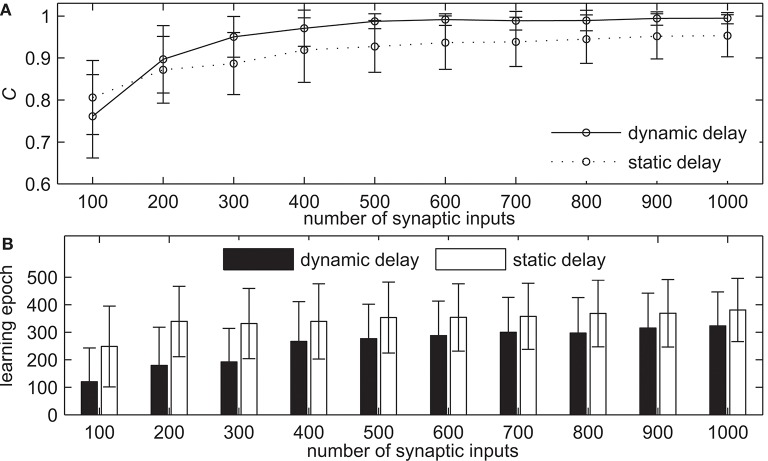
The learning results with the different numbers of synaptic input *N*_*I*_ for the network with dynamic delays and static delays after 500 learning epochs. **(A)** The learning accuracy *C*. **(B)** The learning epochs when the learning accuracy *C* reaches the maximum value.

In this section, we analyze the parameters of network simulation that may influence the learning performance of delay learning algorithm and compare with the network with static synaptic delays on learning performance. The first simulation demonstrates the learning ability of our method with the different numbers of synaptic input *N*_*I*_. The learning results are shown in Figure 7. The *N*_*I*_ increases from 100 to 1, 000 with an interval of 100, while the other settings remain the same. Figure 7A shows the learning accuracy after 500 learning epochs. It can be seen that both the network with dynamic delays and static delays can learn with high accuracy, but the learning accuracy of the network with dynamic delays is higher. The learning accuracy of both two methods increases when *N*_*I*_ increases gradually. For example, the measure *C* = 0.9709 for the network with dynamic delays and *C* = 0.9189 for the network with static delays when *N*_*I*_ = 400. When *N*_*I*_ = 900, the measure *C* = 0.9941 for the network with dynamic delays and *C* = 0.9516 for the network with static delays. Figure 7B shows the learning epochs when the measure *C* reaches the maximum value. From Figure 7B we can see that when *N*_*I*_ increases gradually, the learning epochs of both the network with dynamic delays and static delays are increased slightly, but the learning epochs of the network with dynamic delays are less than that of the network with static delays. When *N*_*I*_ = 400, the mean learning epoch is 266.83 for the network with dynamic delays and 338.89 for the network with static delays. When *N*_*I*_ = 900, the mean learning epoch is 314.95 for the network with dynamic delays and 368.82 for the network with static delays.

**Figure 8:**
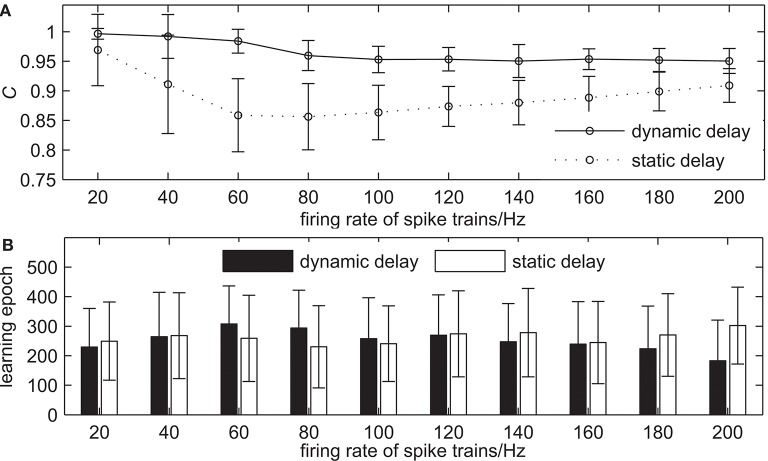
The learning results with the different firing rates of spike trains for the network with dynamic delays and static delays after 500 learning epochs. **(A)** The learning accuracy *C*. **(B)** The learning epochs when the learning accuracy *C* reaches the maximum value.

The second simulation demonstrates the learning ability of our proposed algorithm with the different firing rates of input and desired output spike trains. The learning results are shown in Figure 8. The firing rate of spike trains increases from 20 to 200 Hz with an interval of 20 Hz and the firing rate of input spike trains equals to that of desired output spike trains, while the other settings remain the same. Figure 8A shows the learning accuracy with measure *C* after 500 learning epochs. From Figure 8A we can see that when the firing rate of spike trains increases gradually, the learning accuracy of the network with dynamic delays decreases slightly, while the learning accuracy of the network with static delays decreases first, and then increases slightly, but the learning accuracy of the network with dynamic delays is higher than that of the network with static delays. For example, the measure *C* = 0.9841 for the network with dynamic delays and *C* = 0.8588 for the network with static delays when the firing rate of spike trains is 60 Hz. When the firing rate of spike trains is 140 Hz, the learning accuracy *C* = 0.9504 for the network with dynamic delays and *C* = 0.8801 for the network with static delays. Figure 8B shows the learning epochs when the learning accuracy *C* reaches the maximum value. It can be seen that the learning epochs of the network with dynamic delays are less than that of the network with static delays in the most case. When the firing rate of spike trains is 140 Hz, the mean learning epoch for the network with dynamic delays is 246.98, and 368.82 for the network with static delays.

**Figure 9:**
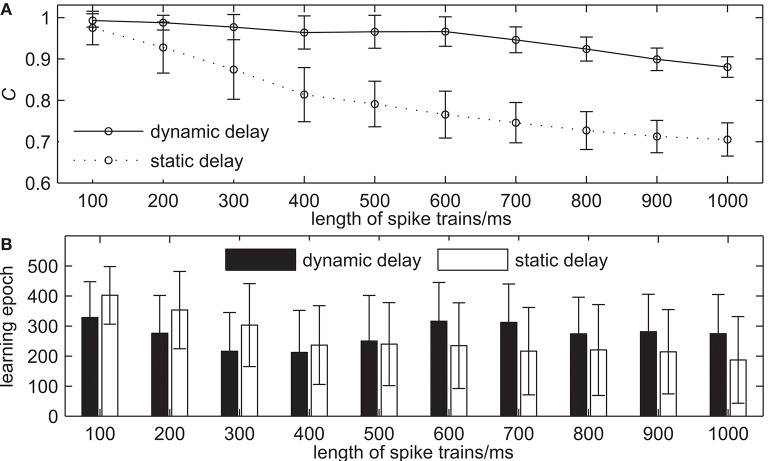
The learning results with the different lengths of spike trains for the network with dynamic delays and static delays after 500 learning epochs. **(A)** The learning accuracy *C*. **(B)** The learning epochs when the learning accuracy *C* reaches the maximum value.

The third simulation demonstrates the learning ability of our proposed algorithm with the different lengths of spike trains. The learning results are shown in Figure 9. The length of spike trains increases from 100 to 1, 000 ms with an interval of 100 ms, while the other settings remain the same. Figure 9A shows the learning accuracy *C* after 500 learning epochs. It can be seen that the learning accuracy of both the network with dynamic delays and static delays decreases when the length of spike trains increases gradually, but the learning accuracy of the network with dynamic delays is higher. For example, the learning accuracy *C* = 0.9767 for the network with dynamic delays and *C* = 0.8743 for the network with static delays when the length of spike trains is 300 ms. When the length of spike trains is 700 ms, the learning accuracy *C* = 0.9461 for the network with dynamic delays and *C* = 0.7460 for the network with static delays. Figure 9B shows the learning epochs when the learning accuracy *C* reaches the maximum value. It can be seen that the learning epochs of the network with dynamic delays are less than that of the network with static delays when the length of spike trains is short. For example, when the length of spike trains is 300 ms, the mean learning epoch for the network with dynamic delays is 215.68, and 302.86 for the network with static delays.

### 3.3. Image Classification

#### 3.3.1. Simulation Setup

**Figure 10:**
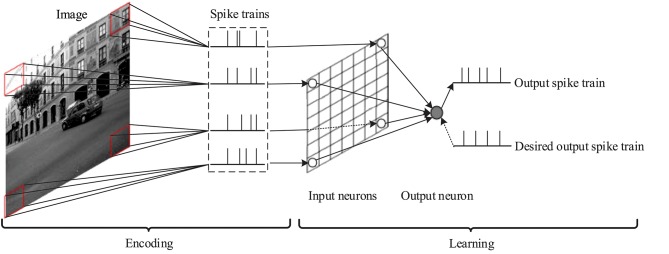
Network structure for image classification.

Here we use the proposed delay learning algorithm to solve an image classification problem, and compare with some other supervised learning algorithms for spiking neurons. The general structure of the network for image classification is shown in Figure 10. It contains 2 functional parts: encoding and learning. In the encoding part, the latency-phase encoding method (Nadasdy, 2009) is used to transform the pixels of the image receptive field into precisely timed spike trains. In the learning part, each spike train corresponding to an input neuron is input into the spiking neural networks. The synaptic weights and delays are learned by the proposed delay learning algorithm. The spiking neural network outputs the target spike pattern for given images.

**Figure 11:**
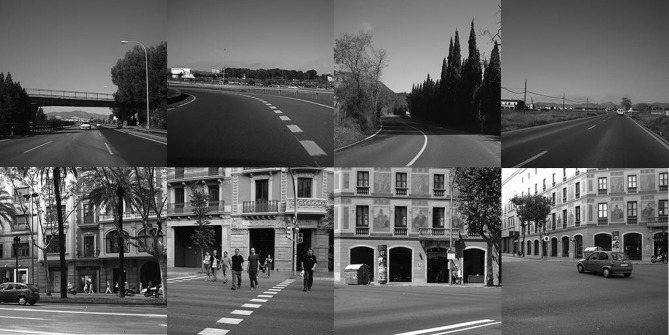
Some images from the LabelMe dataset.

We choose the outdoor road images and the outdoor city street images from the LabelMe dataset (Russell et al., 2008) in the simulation. Each kind of images includes 20 samples, in a total of 40 samples. Figure 11 shows some typical outdoor road images (top) and outdoor city street images (bottom). In our simulation, we choose 10 samples randomly from the outdoor road images and the outdoor city street images respectively (in total 20 samples, 50%) to constitute the training set, while the remaining 20 samples (50%) are constituted the testing set. The original images are converted into 256 × 256 gray images and then encoded into spike trains by the latency-phase encoding. In addition, we need to set the desired output spike trains of two kinds of images. The desired output spike train of the outdoor road images is set as [20, 40, 60, 80] ms, while that of the outdoor city street images is set as [40, 60, 80, 100] ms. The upper limit of learning epochs in the image classification is 50, and each result is averaged over 20 trials.

#### 3.3.2. Learning With Different Sizes of Receptive Field

Table 2 shows the image classification accuracy on the testing set of the LabelMe dataset with different sizes of receptive field. The number of input neurons *N*_*I*_ equals the size of an image divided by the size of receptive field *RF*. The size of receptive field takes 2 × 2, 4 × 4, 8 × 8, 16 × 16, 32 × 32, and 64 × 64 in totals of six values. As seen from the table, with the increasing of *RF*, the testing accuracy of both the network with dynamic delays and static delays are firstly increased, and then decreased. In addition, the testing accuracy of the network with dynamic delays is higher than that of the network with static delays. When the size of the receptive field is 8 × 8, the testing accuracy of both the network with dynamic delays and static delays reached the highest 99.17 and 98.75%, respectively. The receptive field cannot be too large or too small. The appropriate size of the receptive field will obtain higher testing accuracy. The simulation results show that the proposed delay learning algorithm can be applied to image classification problem and achieve high classification accuracy.

**Table 2:** The image classification accuracy on the testing set with different sizes of receptive field.

**RF**	**N_I**	**Dynamic delays**	**Static delays**
2 × 2	16, 384	90.36%±0.09	89.54%±0.07
4 × 4	4, 096	92.68%±0.06	91.39%±0.07
8 × 8	1, 024	99.17%±0.03	98.75%±0.03
16 × 16	256	97.46%±0.05	95.41%±0.05
32 × 32	64	91.40%±0.08	89.73%±0.08
64 × 64	16	90.80%±0.11	85.21%±0.13

#### 3.3.3. Compare With Other Algorithms

**Figure 12:**
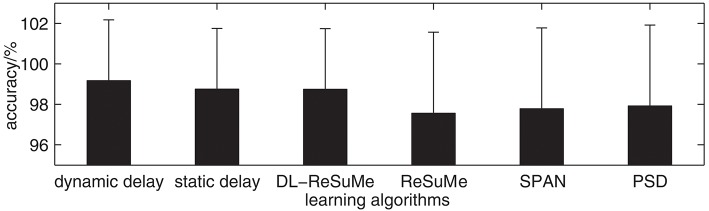
The image classification accuracy of different algorithms.

The ReSuMe algorithm (Ponulak and Kasiński, 2010) has been used to solve the image classification problem (Hu et al., 2013), while the DL-ReSuMe algorithm (Taherkhani et al., 2015a) is a ReSuMe-based delay learning algorithm. In addition, SPAN (Mohemmed et al., 2012) and PSD (Yu et al., 2013) are two typical supervised learning algorithms for spiking neurons based on spike train convolution, which are similar to our proposed learning algorithm. Therefore, we use our proposed learning algorithm and DL-ReSuMe, ReSuMe, SPAN, PSD to solve the image classification problem, and further compare the image classification accuracy of these algorithms. The size of the receptive field is 8 × 8. The resulting image classification accuracy of these algorithms on the testing set is shown in Figure 12. The image classification accuracy of these algorithms on the testing set is 99.17% (dynamic delays), 98.75% (static delays), 98.74% (DL-ReSuMe), 97.56% (ReSuMe), 97.78% (SPAN), and 97.92% (PSD), respectively. It can be seen that all these algorithms can achieve high classification accuracy, but the accuracy of the network with dynamic delays is the highest.

## 4. Discussion

In section 2.2.1, we introduced a supervised learning rule of synaptic weights based on spike train kernels for spiking neurons. The spike train is converted to a unique continuous function through a specific kernel function using the convolution. Then we construct the spike train error function through the convolved continuous functions corresponding to the actual output spike train and desired output spike train, and further deduce the supervised learning rule of synaptic weights by gradient descent method. The learning rule of synaptic weights is finally represented as the form of spike train kernels, which is similar to SPAN (Mohemmed et al., 2012) and PSD (Yu et al., 2013). It can be seen as a general framework of supervised learning algorithms for spiking neurons based on spike train convolution, in which different kernel functions can be used. The derivation of our proposed learning algorithm is independent of the spiking neuron model; it can be theoretically applied to any spiking neuron models. In the training process, the learning rate of synaptic weights is adjusted adaptively according to the firing rate of actual output spike train of neurons.

A new supervised learning rule of synaptic delays based on spike train kernels for spiking neurons is presented in section 2.2.2. The learning rule of synaptic delays is finally represented as the form of spike train kernels, which is similar to the learning rule of synaptic weights. For the sake of simplicity, we use the Laplacian kernel function in the derivation of learning rules. In fact, the general expression of the learning rule of synaptic delays is:

(32) $$\begin{array}{l} \Delta d_{i}=\alpha w_{i}\int_{\Gamma }\left\{\left[f_{s_{d}}\left(t\right)-f_{s_{o}}\left(t\right)\right]\frac{\partial \left[\sum_{f=1}^{N_{i}}\kappa \left(t-t_{i}^{f}-d_{i}\right)\right]}{\partial d_{i}}\right\}dt \end{array}$$

In theory, as long as the kernel function $\kappa \left(t-t_{i}^{f}-d_{i}\right)$ is differentiable to *d*_*i*_, such kernel functions can be used in the delay learning rule. If we choose different kernel functions, then the expression of the partial derivative in Equation (32) is different, and consequently, the expression of Δ*d*_*i*_ is different.

There are some supervised delay learning algorithms for SNNs have been proposed in recent years. The first kind of supervised delay learning algorithms is ReSuMe-based delay learning algorithms (Taherkhani et al., 2015a,b, 2018; Guo et al., 2017). These algorithms merge the delay shift approach and ReSuMe-based weight adjustment (Ponulak and Kasiński, 2010) to enhance the learning performance of the original ReSuMe algorithm. Corresponding to the learning rules of synaptic weights, these algorithms can be regarded as supervised synaptic delay learning algorithms based on synaptic plasticity. The second kind of supervised delay learning algorithms is SpikeProp-based delay learning algorithms (Schrauwen and Van Campenhout, 2004; Matsuda, 2016; Shrestha and Song, 2016). These algorithms provide additional learning rule for the synaptic delays to improve the learning ability of the SpikeProp algorithm (Bohte et al., 2002). Similarly, these algorithms can be regarded as supervised synaptic delay learning algorithms based on gradient descent rule. There are also some other delay learning algorithms (Napp-Zinn et al., 1996; Wang et al., 2012; Hussain et al., 2014; Matsubara, 2017) have been proposed. Our proposed delay learning algorithm employs the spike train kernel to construct the error function, and then deduce the supervised learning rules of synaptic weights and delays. It can be seen as supervised synaptic delay learning algorithms based on spike train convolution. The kernel function is important for this kind of algorithm, in which different kernel functions can lead to different expressions of delay learning rule. It is an open question to consider which kernel function to choose in theory and practical application.

Analysis of the simulations in section 3 indicates that the proposed delay learning algorithm can obtain comparable learning results with different learning parameters. At first, the algorithm is applied to the learning sequences of spikes. The learning results show that the proposed delay learning algorithm can successfully learn the desired output spike train. Then the parameters of synaptic delays are analyzed by simulation of spike train learning. The learning results show that the proposed delay learning algorithm can learn with the different learning rates of synaptic delays and the maximum allowed synaptic delays in a large range. The upper limit of learning epochs is also analyzed. The simulation results show that after 500 learning epochs, the proposed delay learning algorithm can obtain a relatively high learning accuracy. In addition, we analyze the factors that may influence the learning performance and compare with the network of static synaptic delays on learning performance. The simulation results show that the network with dynamic synaptic delays achieved higher learning accuracy and less learning epochs than that of the network with static synaptic delays. When the number of synaptic inputs increases, the learning accuracy of network with dynamic synaptic delays increases. When the firing rate of spike trains or the length of spike trains increases, the learning accuracy of network with dynamic synaptic delays decreases. Finally, we use the proposed delay learning algorithm to solve an image classification problem and archived higher classification accuracy in comparison of other similar supervised learning algorithms for spiking neurons.

The synaptic weight training is the dominant element of supervised learning for SNNs. However, delay training can improve the learning accuracy of SNNs. We tested the learning results of dynamic weights versus static weights under benchmark conditions (Table 1) over 100 trials. The corresponding learning accuracy *C* is shown in Table 3. When both the synaptic delays and weights are static, which means the random initial state of the SNNs, the learning accuracy is *C* = 0.6123. When the synaptic weights are static while the synaptic delays are dynamic, the learning accuracy is *C* = 0.6528. It shows that the dynamic delays can improve learning accuracy. When the synaptic weights are dynamic while the synaptic delays are static, the learning accuracy is *C* = 0.9274, which is significantly higher than that of the network with static weights. When both the synaptic delays and weights are dynamic, the learning accuracy *C* = 0.9874 is height. In summary, both the synaptic weights and delays have an impact on network training, but the impact of synaptic weights is greater. Delay training cannot replace weight training but can improve the learning accuracy of SNNs.

**Table 3:** Learning accuracy *C* of the delay learning algorithm.

	**Static weights**	**Dynamic weights**
Static delays	0.6123 ± 0.0862	0.9274 ± 0.0616
Dynamic delays	0.6528 ± 0.0739	0.9874 ± 0.0178

## 5. Conclusion

In this paper, we introduced a new supervised delay learning algorithm based on spike train kernels for spiking neurons. In this method, both the synaptic weights and the synaptic delays can be adjusted. We applied the proposed algorithm to a series of spike train learning experiments and an image classification problem to demonstrate the learning ability of spike train spatio-temporal pattern, and compared with the network with static synaptic delays on learning performance. Simulation results show that both the network with dynamic delays and static delays can successfully learn a random spike train and solve image classification problem, and the network with dynamic delays has higher learning accuracy and less learning epochs than that of the network with static delays.

Generally speaking, the more complex a neural network is, the more powerful its computing power is. The proposed supervised learning algorithm of synaptic delays in this paper can be applied only for a single layer SNNs, which limits the computing power of SNNs. We have proposed two supervised learning algorithms of synaptic weights for multi-layer feed-forward SNNs (Lin et al., 2017) and recurrent SNNs (Lin and Shi, 2018) based on inner products of spike trains. In the future work, we will extend the proposed delay learning algorithm to multi-layer feed-forward SNNs and recurrent SNNs to solve more complex and practical spatio-temporal pattern recognition problems.

## Data Availability

Publicly available datasets were analyzed in this study. This data can be found here: http://labelme.csail.mit.edu/Release3.0/.

## Author Contributions

XW wrote the paper and performed the simulations. XL conceived the theory and designed the simulations. XD discussed about the results and analysis, and reviewed the manuscript. All authors helped with developing the concepts, conceiving the simulations, and writing the paper.

### Conflict of Interest Statement

The authors declare that the research was conducted in the absence of any commercial or financial relationships that could be construed as a potential conflict of interest.
